# Descriptive epidemiology of selected birth defects, areas of Lombardy, Italy, 1999

**DOI:** 10.1186/1478-7954-5-4

**Published:** 2007-05-25

**Authors:** Giovanna Tagliabue, Roberto Tessandori, Fausta Caramaschi, Sabrina Fabiano, Anna Maghini, Andrea Tittarelli, Daniele Vergani, Maria Bellotti, Salvatore Pisani, Maria Letizia Gambino, Emanuela Frassoldi, Enrica Costa, Daniela Gada, Paolo Crosignani, Paolo Contiero

**Affiliations:** 1 Cancer Registry Division, Istituto Nazionale per lo Studio e la Cura dei Tumori, Milan, Italy; 2 Local Health Authority of Sondrio Province, Sondrio, Italy; 3 Local Health Authority of Mantova Province, Mantova, Italy; 4 Cardiology Service, Presidio Ospedaliero Vittore Buzzi, Milano, Italy; 5 Department of Obstetrics and Gynecology, DMCO San Paolo, University of Milan, Italy; 6 Local Health Authority of Varese Province, Varese, Italy

## Abstract

**Background:**

Birth defects are a leading cause of neonatal and infant mortality in Italy, however little is known of the etiology of most defects. Improvements in diagnosis have revealed increasing numbers of clinically insignificant defects, while improvements in treatment have increased the survival of those with more serious and complex defects. For etiological studies, prevention, and management, it is important to have population-based monitoring which provides reliable data on the prevalence at birth of such defects.

**Methods:**

We recently initiated population-based birth defect monitoring in the Provinces of Mantova, Sondrio and Varese of the Region of Lombardy, northern Italy, and report data for the first year of operation (1999). The registry uses all-electronic source files (hospital discharge files, death certificates, regional health files, and pathology reports) and a proven case-generation methodology, which is described.

The data were checked manually by consulting clinical records in hospitals. Completeness was checked against birth certificates by capture-recapture. Data on cases were coded according to the four-digit malformation codes of the International Classification of Diseases, Ninth Revision (ICD-9). We present data only on selected defects.

**Results:**

We found 246 selected birth defects in 12,008 live births in 1999, 148 among boys and 98 among girls. Congenital heart defects (particularly septal defects) were the most common (90.8/10,000), followed by defects of the genitourinary tract (34.1/10, 000) (particularly hypospadias in boys), digestive system (23.3/10,000) and central nervous system (14.9/10,000), orofacial clefts (10.8/10,000) and Down syndrome (8.3/10,000). Completeness was satisfactory: analysis of birth certificates resulted in the addition of two birth defect cases to the registry.

**Conclusion:**

This is the first population-based analysis on selected major birth defects in the Region. The high birth prevalences for septal heart defect and hypospadias are probably due to the inclusion of minor defects and lack of coding standardization; the latter problem also seems important for other defects. However the data produced are useful for estimating the demands made on the health system by babies with birth defects.

## Background

Birth defects are relatively common, affecting 3% to 5% of live-births in the United States [1] and 2.1% in Europe [2]. For more than two decades, birth defects have been the leading cause of infant mortality in the US [3]. Birth defects are also the leading cause of infant mortality in the Region of Lombardy, northern Italy. In 1998, 2800 infants in the Region of Lombardy died in the first year of life; of these 852 had birth defects (16/10,000) [4]. The morbidity and disability experienced by surviving children also has a major public health impact.

Around 40% to 60% of birth defects are of unknown origin; 20% are attributed to a combination of heredity and other factors; 7.5% to single gene mutations; 6% to chromosome abnormalities; and 5% to maternal illnesses, such as diabetes or infection, or use of anticonvulsant or other drugs [5-7].

The prevalence rate of birth defects is increasingly used as an indicator of exposure to teratogens of various kinds, particularly pesticides but also pharmaceuticals [8].

Registries and surveillance systems are the only way to obtain population-based data on birth defects, since birth certificates have been shown to be inadequate [9]. We set up a registry in 1999, covering part of the Region of Lombardy, to monitor the annual prevalence rates of birth defects. The aims of the present study are (a) to briefly describe the structure of the system used to gather case information and generate birth defect data emphasizing features that ensure data quality, (b) to present a preliminary analysis of prevalence rates for selected birth defects in children born in 1999 in the Provinces of Mantova, Sondrio and Varese (Region of Lombardy); and (c) to assess the quality of data produced by performing a completeness evaluation using birth certificates for comparison, and comparing rates with those published by long established birth defect registries.

## Methods

A birth defect is defined by the registry as a structural or chromosomal anomaly present at birth and recognized during the first year of life. The registry records all birth defects in live births. However, the present analysis concerns selected defects: those considered major by Rasmussen et al [10] and the International Clearinghouse for Birth Defects [11], except that limb deficiencies are not considered, due to problems with diagnosis and coding in our dataset.

### Case definition

For inclusion in the registry

• The child must be born live

• The mother must be resident in Mantova, Sondrio or Varese Province when the child is born.

• The defect must be on the ICD-9 list (International Classification of Diseases, Ninth Revision, 1979, WHO Geneva)

• The defect must be diagnosed before the child's first birthday

### Case ascertainment

Population-based case ascertainment employs automated registration from electronic mortality files, hospital discharge files, and pathology files. Mortality files are provided by the local health authorities of the three provinces. Hospital discharge files, provided by the health department of the Region of Lombardy, contain records from all hospitals in Italy. This is important as many congenital anomalies are diagnosed prenatally and the women give birth at specialist hospitals outside the study areas. Pathology reports are obtained from pathology departments in the three provinces and from some outside. To ensure maximum completeness, information for a given calendar year is collected up to December of the succeeding year (data registration may occur in the year following diagnosis).

The Birth Defect Registry uses the proven Open Registry software [12-16] to generate cases. The steps in this procedure are as follows:

• Electronic files are extracted from the source information systems, standardized, and loaded into Open Registry tables.

• Records pertaining to a single individual from various sources are linked by proven linkage algorithms [17]

• Birth defect records are generated from the tables.

• Birth defect records are manually checked against paper sources to eliminate false positives and check diagnosis accuracy.

#### Extraction and standardization of data

Open Registry loads the three data source files and in addition loads a fourth electronic data file, the Social Security List (SSL) of the Lombardy region. The SSL contains the name, surname, date of birth, sex, residence code and a unique social security number, of all persons living in the Lombardy Region. Registry staff launch queries to check that all the necessary data are available and have the correct format (for example the program checks that the ICD-9 disease code has four digits). Routines standardize the data to a single format and pass them automatically to an Oracle table. Once loaded into Oracle tables, automatic checks for consistency are performed (e.g. number of records is within expected range from a given hospital). Open Registry routines then scan the records and select those with disease codes indicating birth defects. Specifically, the software selects records with ICD-9 codes from 740 to 759 inclusive.

#### Data linkage

The next step is to link the records selected in the previous step so as to generate a single record for each case. To facilitate this Open Registry makes use of the SSL with a unique social security number for each case. The social security number renders data linkage straightforward but if it is missing or erroneous, other identifiers can be used. Open Registry incorporates the proven Epilink record linkage software [17] that employs a combined deterministic and statistical approach to linking records. The social security number is transferred to every successfully linked record. Because nearly all SSL records contain a residence code, the linkage process also identifies (theoretically) all residents in the Registry Provinces. Records not linked to the SSL are checked manually by telephoning the Residence Office of the Municipality. The use of a file such as the SSL is recommended by the Guidelines for Conducting Birth Defects Surveillance [18].

#### Case generation and verification

Following data linkage, Open Registry automatically generates a single personal data record for each case, together with one or more linked birth defect records, each with details of a single birth defect. A second birth defect record will be present if a given case has second birth defect. The disconnection between personal and clinical information helps ensure privacy, and is required by Italian legislation.

Each birth defect record is then checked manually against paper records from the hospitals that performed the diagnosis in order to eliminate false positives and correct any discrepant birth defect code.

Finally, all cases are reviewed by a pediatrician specialized in pathology and genetics, to ensure recognition of syndromes in children with multiple anomalies.

### Statistical methods

We used the capture-recapture technique [19] to estimate the true number of birth defects in the Lombardy Birth Defect Registry, using birth certificates as independent source of cases compared to all other registry sources. The estimated total number was the best estimate of total birth defect prevalence. Confidence intervals were calculated assuming Poisson distributions.

### Ethical approval

The Lombardy birth defect registry project was approved by the Ethical Committee of the National Cancer Institute. The project was then approved by the Privacy Guarantee Authority (Italian Authority protecting confidentiality). Strict safeguards have been established to prevent unauthorized use of records. The highest level of confidentiality is maintained for all identifying information.

### Study areas

Mantova has an area of 2,339 sq km, population of 376,000 and birth rate 8.4/1,000; Sondrio has an area of 3,212 sq km, population 177,500 and birth rate 9.2/1,000; Varese has an area of 1,198 sq km, population 821,000 and birth rate 9.2/1,000. The three Provinces contain 15% of the total population of the Region of Lombardy, whose birth rate is around 9.6/1,000 per year.

## Results

### Birth defects

A total of 246 congenital malformations were identified among the 12,008 live births in 1999. Among boys there were 148 malformations, giving a prevalence rate at birth of 241.9/10,000, and among girls there were 98 birth defects giving a birth prevalence rate of 166.4/10,000. Prevalence rates at birth for selected congenital malformations are shown in Tables 1 and 2.

**Table 1:** Prevalences at birth of selected defects in girls born the Provinces of Mantova, Sondrio and Varese, Region of Lombardy, 1999

Birth defects	Number of cases	Birth prevalence/10,000 and 95%CI
Anencephaly	0	0 (0–6.3)
Spin bifida	1	1.7 (0–9.5)
Encephalocele	0	0 (0–6.3)
Microcephaly	1	1.7 (0–9.5)
Arhinencephaly/holoprosencephaly	4	6.8 (1.9–17.4)
Hydrocephaly	4	6.8 (1.9–17.4)
Buphtalmos	1	1.7 (0–9.5)
Congenital cataract	2	3.4 (0.4–12.3)
Anotia/microtia	1	1.7 (0–9.5)
Transposition of great vessels	4	6.8 (1.9–17.4)
Tetralogy of Fallot	2	3.4 (0.4–12.3)
Ventricular septal defect	42	71.3 (51.4–96.4)
Atrial septal defect	4	6.8 (1.9–17.4)
Endocardial cushion defects	3	5.1 (1.1–14.9)
Ebstein's anomaly	1	1.7 (0–9.5)
Hypoplastic left heart syndrome	0	0 (0–6.3)
Coarctation of aorta	3	5.1 (1.1–14.9)
Choanal atresia, bilateral	0	0 (0–6.3)
Cleft palate without cleft lip	1	1.7 (0–9.5)
Cleft lip with or without cleft palate	2	3.4 (0.4–12.3)
Oesophageal atresia/stenosis	4	6.8 (1.9–17.4)
Congenital hypertrophic pyloric stenosis	1	1.7 (0–9.5)
Small intestine atresia/stenosis	0	0 (0–6.3)
Anorectal atresia/stenosis	1	1.7 (0–9.5)
Hirschsprung's disease	1	1.7 (0–9.5)
Anomalies of intestinal fixation	0	0 (0–6.3)
Hypospadias		
Indeterminate sex	0	0 (0–6.3)
Renal agenesis	1	1.7 (0–9.5)
Cystic kidney	3	5.1 (1.1–14.9)
Bladder exstrophy	0	0 (0–6.3)
Polydactyly and syndactyly	4	6.8 (1.9–17.4)
Total limb reduction defects	Not analysed	Not analysed
Diaphragmatic hernia	1	1.7 (0–9.5)
Omphalocele	0	0 (0–6.3)
Gastroschisis	0	0 (0–6.3)
Trisomy 13	0	0 (0–6.3)
Trisomy 18	0	0 (0–6.3)
Down syndrome	6	10.2 (3.7–22.2)
Prader Willi syndrome	0	0 (0–6.3)
Total	98	166.4

**Table 2:** Prevalences at birth of selected defects in boys born in the Provinces of Mantova, Sondrio and Varese, Region of Lombardy, 1999

Birth defect	Number of cases	Birth prevalence/10,000 and 95% CI
Anencephaly	0	0 (0–6)
Spina bifida	1	1.6 (0–9.1)
Encephalocele	0	0 (0–6)
Microcephaly	3	4.9 (1–14.3)
Arhinencephaly/Holoprosencephaly	1	1.6 (0–9.1)
Hydrocephaly	3	4.9 (1–14.3)
Buphtalmos	0	0 (0–6)
Congenital cataract	2	3.3 (0.4–11.8)
Anotia/microtia	1	1.6 (0–9.1)
Transposition of great vessels	4	6.5 (1.8–16.7)
Tetralogy of Fallot	1	1.6 (0–9.1)
Ventricular septal defect	28	45.6 (30.4–66.2)
Atrial septal defect	9	14.7 (6.7–27.9)
Endocardial cushion defects	1	1.6 (0–9.1)
Ebstein's anomaly	0	0 (0–6)
Hypoplastic left heart syndrome	1	1.6 (0–9.1)
Coarctation of aorta	6	9.8 (3.6–21.3)
Choanal atresia, bilateral	0	0 (0–6)
Cleft palate without cleft lip	5	8.2 (2.7–19.1)
Cleft lip with or without cleft palate	5	8.2 (2.7–19.1)
Oesophageal atresia/stenosis	1	1.6 (0–9.1)
Congenital hypertrophic pyloric stenosis	12	19.6 (10.1–34.3)
Small intestine atresia/stenosis	2	3.3 (0.4–11.8)
Anorectal atresia/stenosis	1	1.6 (0–9.1)
Hirschsprung's disease	3	4.9 (1–14.3)
Anomalies of intestinal fixation	2	3.3 (0.4–11.8)
Hypospadias	31	50.7 (34.4–71.9)
Indeterminate sex	0	0 (0–6)
Renal agenesis	3	4.9 (1–14.3)
Cystic kidney	3	4.9 (1–14.3)
Bladder exstrophy	0	0 (0–6)
Polydactyly and syndactyly	11	18 (9–32.2)
Total limb reduction defects	Not analysed	Not analysed
Diaphragmatic hernia	3	4.9 (1–14.3)
Omphalocele	0	0 (0–6)
Gastroschisis	0	0 (0–6)
Trisomy 13	0	0 (0–6)
Trisomy 18	0	0 (0–6)
Down syndrome	4	6.5 (1.8–16.7)
Prader Willi syndrome	1	1.6 (0–9.1)
Total	148	241.9

### Congenital malformations of the central nervous system

One case of spina bifida with hydrocephalus (female) and one without hydrocephalus (male) were registered with associated prevalence rates at birth of 1.7/10,000 for females and 1.6/10,000 for males. Reduction deformities of brain were reported for four girls (6.8/10,000) and one boy (1.6/10,000). Congenital hydrocephalus was reported for 3 boys and 4 girls (4.9 and 6.8/10,000). No cases of anencephalus or encephalocele were reported.

### Congenital anomalies of eye, ear, face and neck

Five congenital anomalies of eye were recorded in girls (8.5/10,000) and five in boys (8.2/10,000). The most common were congenital cataract (two in boys and two in girls giving prevalences of 3.3 and 3.4/10,000 respectively), and five congenital anomalies of eyelid/lachrymal system (8.5/10,000, data not shown). One girl had congenital glaucoma (1.7/10,000). Congenital cataracts are reported to account for 30% of congenital eye malformations in live babies, and have an estimated incidence of 1 to 10,000 births [20]. In boys, seven head and neck defects were found, one being a branchial fistula (1.6/10,000). In girls, three head and neck anomalies were registered, two of which were branchial fistulae (3.4/10,000) (data not shown).

### Congenital anomalies of the cardiovascular system

Congenital anomalies of the heart affected more infants born in the study area than any other type of birth defect: 59 (49.1/10,000) in girls and 50 in boys (41.6/10,000). The two most common types of heart malformations were ventricular septal defect (VSD) and atrial septal defect (ASD). There were 4 ASD cases (6.8/10,000) and 42 VSD cases (71.3/10 000) in girls, and 9 ASD cases (14.7/10,000) and 28 VSD cases (45.8/10,000) in boys.

Four cases of transposition of the great arteries were registered in girls (6.8/10,000) and four in boys (6.5/10,000). We found nine cases of aortal coarctation, six in boys (9.8/10,000) and three in girls (5.1/10,000); two cases of tetralogy of Fallot (3.4/10, 000) in girls and one (1.6/10,000) in a boy; three female cases of endocardial cushion defect (5.1/10,000) and only one case in boys (1.6/10,000); and one female case of Ebstein's anomaly (1.7/10,000).

### Congenital anomalies of lip and palate

We found 13 cases of orofacial cleft, three in girls (5.1/10,000) – one with cleft palate, one with cleft lip and one with both anomalies – and 10 in boys – five with cleft palate (8.2/10,000), two with cleft lip (3.3/10,000) and three with both (4.9/10, 000).

### Congenital anomalies of the digestive system

Esophageal atresia and esophagotracheal fistula usually occur together, but in about 10% of cases they occur separately. Four girls (6.8/10,000) had these defects: two had atresia with fistula; one had atresia only and one – fistula only. One boy (1.6/10,000) had esophageal atresia alone.

One girl (1.7/10,000) and 12 boys (19.6/10,000) had hypertrophic stenosis of pylorus, a sex ratio consistent with that in the literature [21].

We found two boys with small intestine atresia (3.3/10,000) and two with intestinal malrotation (3.3/10,000), one of which associated with congenital duodenal stenosis.

One boy (1.6/10,000) and one girl (1.70/10,000) had anal agenesis; in the latter rectovestibular fistula was associated: she underwent surgical correction soon after birth.

Hirschsprung's disease was diagnosed in three boys (4.9/10,000) and one girl (1.7/10, 000).

### Congenital anomalies of the urogenital system

Hypospadias is the most common developmental anomaly of male urogenital organs. The mild forms (distal, coronal, and glandular hypospadias) are common, while only severe hypospadias (opening to penile shaft, scrotum or perineum) are usually registered. We included both severe and mild forms of hypospadias, a total of 31 cases (50.7/10,000). One case of epispadia was found (data not shown).

Three boys had renal agenesis, (4.9/10,000), one with a family history of Potter's syndrome. Three boys had cystic kidney disease (4.9/10,000). Among girls, one had renal agenesis (1.7/10,000) and three had cystic kidney disease (5.1/10,000).

### Congenital anomalies of limbs and musculoskeletal system

Structural limb anomalies include dysplasias, reduction defects, and duplication defects with supernumerary limb elements. Most human limb defects appear to have a multifactorial etiology. Among boys, we had four cases of polydactyly (one bilateral on hands, two monolateral on hand, and one monolateral on foot) and seven of syndactyly (overall birth prevalence 18/10,000).

Among girls we found three cases of polydactyly (5.1/10,000) (one on hands bilaterally, one on monolateral hand, one on foot bilaterally). One girl had left hallux agenesis (1.7/10,000, data not shown) and another girl had osteochondrodystrophy (1.7/10,000, data not shown).

### Congenital diaphragmatic hernia

In congenital diaphragmatic hernia one of the pericardioperitoneal canals fails to close so the developing abdominal viscera to bulge into the pleural cavity. We had four cases of this defect, three boys (4.9/10,000) and one girl (1.7/10,000). One of the boys had associated lung hypoplasia and the girl had associated lung agenesis. Two of the boys died soon after birth of severe pulmonary insufficiency.

### Chromosomal anomalies

Four boys (6.5/10,000) and six girls (10.2/10,000) had trisomy 21 or Down syndrome, the most commonly reported congenital autosomal anomaly.

One boy had Prader Willi Syndrome (1.6/10,000) resulting from deletion of a region of human chromosome 15 (15q11-q13). This syndrome is diagnosed by DNA methylation analysis, fluorescence in situ hybridisation and identification of DNA polymorphisms [22].

Table 3 compares the birth prevalence of the 40 selected birth defects in the Lombardy area with those in Georgia USA, Hawaii USA (as presented in Congenital Defects Program) [23] and Finland [11] (as presented in the Clearinghouse Birth Defects program). Table 4 summarizes the working methods of the three birth defect registries in comparison to the Lombardy registry.

**Table 3:** Comparison of overall birth prevalences (per 10,000) of selected defects in Lombardy (1999), Finland (1998), Georgia and Hawaii (1995–1999).

		Lombardy	Finland*	Georgia°	Hawaii°
Number of live births		12 008	57 108	216 009	89 079
Defects					
Total number		246	591	4836	1878
Total prevalence (/10,000)		204.9	103.5	223.9	233.6
Prevalence of individual defects (/10,000)					
					
1	Anencephaly	0	0.2	4.2	3.8
2	Spin bifida	1.7	3.2	4.4	5.2
3	Encephalocele	0	0.3	2.2	2.6
4	Microcephaly	3.3	1.6	8.4	8.5
5	Arhinencephaly/holoprosencephaly	4.2	0.9	Not reported	Not reported
6	Hydrocephaly	5.8	3.2	8.0	11.6
7	Buphtalmos	0.83	Not reported	Not reported	Not reported
8	Congenital cataract	3.3	Not reported	1.9	1.8
9	Anotia/microtia	1.7	4.7	1.4	3.6
10	Transposition of great vessels	6.7	4.0	5.4	5.5
11	Tetralogy of Fallot	2.5	3.3	4.5	3.1
12	Ventricular septal defect	58.3	Not reported	35.6	43.4
13	Atrial septal defect	11	Not reported	25.3	22.8
14	Endocardial cushion defect	3.3	Not reported	5.5	2.6
15	Ebstein's anomaly	0.8	Not reported	0.7	0.9
16	Hypoplastic left heart syndrome	0.8	4.7	3.2	1.5
17	Coarctation of aorta	7.5	8.2	5.6	2.5
18	Choanal atresia, bilateral	0	0.9	1.8	1.2
19	Cleft palate without cleft lip	5.0	16.3	6.5	7.9
20	Cleft lip with or without cleft palate	5.8	8.0	9.3	14.5
21	Oesophageal atresia/stenosis	4.2	3.3	2.0	1.9
22	Congenital hypertrophic pyloric stenosis	10.8	Not reported	13.9	7.4
23	Small intestine atresia/stenosis	1.7	0.7	Not reported	Not reported
24	Anorectal atresia/stenosis	1.7	4.7	3.5	4.8
25	Hirschsprung's disease	3.3	Not reported	2.1	2.2
26	Anomalies of fixation	1.7	Not reported	Not reported	Not reported
27	Hypospadias	25.8	2.6	35.1	29.6
27	Epispadia	Not analysed	0.35	With hypospadia	With hypospadia
28	Indeterminate sex	0	0.3	Not reported	Not reported
29	Renal agenesis	3.3	0.7	4.4	5.6
30	Cystic kidney	5.0	3.3	Not reported	Not reported
31	Bladder exstrophy	0	0.2	0.1	0.6
32	Polydactyly and syndactyly	12.5	2.8	Not reported	Not reported
33	Total limb reduction defects	Not analysed	5.6	5.8	4.7
34	Diaphragmatic hernia	3.3	2.1	2.0	2.6
35	Omphalocele	0	2.1	2.3	3.5
36	Gastroschisis	0	1.7	1.6	4.4
37	Trisomy 13	0	0.9	1.6	2.6
38	Trisomy 18	0	1.9	2.7	6.5
39	Down syndrome	8.3	10.5	12.4	16.0
40	Prader Willi syndrome	0.8	Not reported	Not reported	Not reported

**°Congenital Malformations Surveillance Report. A report from the National Birth Defects Prevention Network**. *Teratology *2002, 66 **Suppl **1:S1-219

*Surveillance and Research.

**Table 4:** Characteristics of the birth defect registries used for comparison with Lombardy birth defects registry

**Characteristic**	**Finland**	**Georgia**	**Hawaii**	**Lombardy**
Type of registry	Population-based	Population-based	Population-based	Population-based
Size (births/year)	58,000	50,019	20,636	12,008
Time for diagnosis	Up to 1 y	Up to 6 y	Up to 1 y	Up to 1 y
Compulsory	Yes	Yes	Yes	No
Defects in abortion/terminations considered?	Yes	Yes	Yes	Not yet
Case ascertainment	Passive	Active	Active	Passive
**Sources:**				
Hospital reports	Yes	Yes	Yes	Yes
Pathology reports	Yes	Yes	Yes	Yes
Cytogenetic lab reports	Yes	Yes	Yes	Yes
Other registries	National registers of abortions and births	No	No	No
Hospital discharges	Yes	Yes	Yes	Yes
Death certificates	Yes	Yes	Yes	Yes
Birth certificates	Yes	Yes	Yes	Yes
Fetal death certificates	No	Yes	Yes	No

### Completeness

The completeness analysis was conducted only on babies born in Varese Province because birth certificates from the other two areas were not available. We had 398 birth defects from Varese, considering all malformations, not only the selected ones for the analysis, but birth certificates identified only 25 defects, including 2 not contained in the registry. Using the capture-recapture method we estimated that there were 431 malformations in Varese (Table 5) so that the sensitivity of our system was 92%.

**Table 5:** Distribution of found and missed birth defects in the Lombardy Birth Defect Registry vs. birth certificates

	Birth defects identified by Registry		
Birth defects identified by birth certificates	Yes	No	Totals
Yes	23	2	25
No	375	31*	406
Totals	398	33	431*

* The number of cases missed by both sources (31) and the total number of birth defects (431) were estimated by the capture-recapture method.

The addition of the two birth certificate cases (a case of cryptorchidism and one of unilateral choanal atresia) did not change our prevalence data as these are not among the selected malformations considered in this analysis.

## Discussion

Use of birth certificates to check completeness added two new cases to the Lombardy birth defects registry. The quality and the completeness of birth certificates is known to vary considerably [24]. We found a large number of false positives and also massive under-reporting, which shows that birth certificates are not a reliable single source for ascertaining congenital malformations [24]. However their availability allowed us to perform a natural experiment to assess our registration system and identify weaknesses in data collection [25].

We discuss our birth defects prevalence findings in relation to those from three other birth defect registries: Georgia USA [23], Hawaii [23] and Finland [11]. There are several reasons for this. All three are large (national or state) long-established population-based national registries, with a history of quality scientific publications. They also use differing data collection methods and cover geographically disparate areas.

The overall prevalence at birth of the 40 selected congenital defects in our study area in 1999 was 204.9/10,000. This compares with 223.9/10,000 for the birth defects registry of Georgia USA in 1995–1999 [23], 233.6/10,000 for the registry of Hawaii [23] from 1995 to 1999, and 103.5/10 000 for birth defects recorded in Finland in 1998 [11].

Cardiovascular system defects were by far the most common birth defects in our population. Prevalence rates for these defects were grossly similar to rates published previously [23], except for ventricular septal defects (VSD), for which we found particularly high overall rates (58.3/10,000). The latter are nonetheless fairly comparable with those reported by Florida (41.6/10,000), Georgia (35.6/10,000) and Hawaii (43.4/10,000) [23]. The elevated prevalence of VSD in our study areas is probably due to over-diagnosis: routine use of echocardiography on virtually all newborn infants has resulted in the identification of large numbers of small and clinically unimportant VSDs. Subsequent echocardiography results in the inclusion of clinically unimportant defects (that close spontaneously). Progress in clinical management and more frequent prenatal diagnosis have reduced neonatal mortality, increasing the prevalence at birth of these defects, while generally only severe congenital heart defects diagnosed *in utero *are aborted [26]. These findings indicate a strong need to standardize both diagnostic and registration criteria for congenital heart malformations, as also suggested by Hofmann [27].

We found rates of 6.7/10,000, 3.3/10,000 and 7.5/10,000 for transposition of great arteries, endocardial cushion defects, and coarctation of aorta, respectively. These figures compare with lower birth prevalences for transposition of great arteries in Finland (4.0/10,000; endocardial cushion defects not reported), and a higher rate for endocardial cushion defect (5.5/10,000) but lower rates for transposition of great arteries (5.4/10,000) and coarctaction of aorta (5.6/10,000) in Georgia. In cases of complete transposition, the aorta arises from the right ventricle and the pulmonary artery arises from the left ventricle, and there is an associated ventricular septal defect, which in all eight of our registered cases was large. Botto et al. [28] observed an increase birth prevalence of atrial and ventricular defects from 1968 to 1995 in the population of Atlanta but no change in the rate of grand artery transpositions.

We found low birth prevalence rates for tetralogy of Fallot (2.5/10,000) and hypoplastic left heart syndrome (0.8/10,000) compared with the corresponding figures of 3.3/10,000 and 4.7/10,000 in Finland, 4.5/10,000 and 3.2/10,000 in Georgia, and 3.1/10,000 and 1.5/10,000 in Hawaii. Our rate of 2.5/10,000 is comparable with other European prevalence rates of 2.56/10,000 and 2.45/10,000 for Central-Eastern France and Sweden respectively [29]. The study by Hoffman [27] reviewed 41 studies on cardiac malformation and found prevalence rates for tetralogy of Fallot ranging from 2.91/10,000 to 5.77/10,000, with a mean of 4.21/10,000.

The birth prevalence of hypospadias varies widely (2.0–39.7/10,000) [30]. We found very high rates of hypospadias in accord with data from Georgia (35.1/10,000) [23] but in stark contrast to the low figure of 2.6/10,000 from Finland [11].

The study by Pierik et al. [31] addressed the highly variable hypospadias rates in Holland, studying a cohort of all newborn in Rotterdam over a two year period. They found a high rate (38/10,000) which is comparable to ours. They concluded that the variation in hypospadias birth prevalence in Holland is due both to differences in case ascertainment and real geographic variation.

The comparability of reported rates for hypospadias is poor, however there may be an important underlying geographic variation which cannot be properly addressed until ascertainment and diagnostic criteria (inclusion or exclusion of minor cases) are standardized [30]. Hypospadias could be related to exposure to antiandrogenic factors that interfere with androgen activity [32]. There is also evidence that increases in these birth defects are linked to increased risk of testicular cancer, both of which may be related to exposure to environmental substances having estrogen-like effects [33]. Genetic factors may also predispose to the development of hypospadias [34].

Our overall figure for Down syndrome was 8.3/10,000. Other published population-based rates for this syndrome are higher [19] (12.4–22.2/10,000). Down syndrome can be diagnosed relatively easily prior to birth by measuring alphafetoprotein, human chorionic gonadotropin and unconjugated estriol in fetal serum, detecting a thickened nuchal fold on fetal ultrasound, and by cytogenetic analysis. Thus, it is likely that many fetuses with Down syndrome are aborted electively and that variable abortion rates contribute to the variable prevalence at birth rates. This conclusion is supported by data from the Strasbourg birth defect registry, which reported the low rate of 2.2/10,000 live births in 1998. This rate however became 19.7/10,000 when aborted fetuses were considered [11]. The number of Down syndrome fetuses has increased as the mean age of pregnant women has increased [35].

With regard to nervous system defects, the prevalence rate for spina bifida in our population was lower (1.7/10,000) than found in other registries (3.2–5.2/10,000). For hydrocephalus the combined male-female rate was 5.8/10,000 in our registry, and varied from 3.2 to 11.6 in the other population-based registries [23]. The reasons for these wide variations in rates for nervous system defects are not known, but it is likely that variations in rates of elective terminations contribute substantially. No cases of anencephalus or encephalocele were found, probably in relation to improved prenatal diagnosis and elective termination. Recent surveillance data on neural tube defects show they are diagnosed prenatally in about 80% of cases [36]. In the US, 20–30% of fetuses with neural tube are terminated [37]. Folate intake also varies between populations, and lack of this nutrient is known to cause such defects [38].

Facial clefts are a heterogeneous group of easy-to-recognize non-fatal birth defects [39]. They are reported as the most common congenital facial abnormalities, with prevalence at birth estimated at 10/10,000 [40] and in the range 6–17 per 10,000 Caucasian births, 4/10,000 for African-Americans and 17/10,000 for Japanese [41]. Our population-based data for facial cleft (overall birth prevalence 10.8/10,000) are in line with these estimates but considerably lower than those for Georgia, (15.8/10,000) and Finland (24.3/10,000). We found that they were about twice as common in boys than girls, which is consistent with other published data [40]. Facial clefts are considered to be etiologically heterogeneous. A small proportion occur as a part of recognizable pattern of malformations or have a genetic etiology [42], while epidemiologic data suggest that exogenous factors contribute to these conditions. Maternal factors that have been studied for their influence on cleft risk include smoking, alcohol consumption, medication use, environmental chemicals and nutritional factors, but none appear to explain a significant proportion of the population burden of these anomalies [43]. Geographic differences in birth prevalence for these anomalies probably reflect differences in maternal life style and exposure to environmental causative factors.

The birth prevalence for esophageal atresia (4.2/10,000) was similar in our study to that reported in Finland (3.3/10,000), whereas that of small intestine atresia was higher (1.7/10,000) in our population than in the Finnish (0.7/10,000). Prevalence in Georgia, (1968 to 1989) has been reported as 2.8/10,000 overall, but higher in black infants (3.7/10,000) [44].

Hirschprung's disease is relatively easy to diagnose, so higher birth prevalence in our population (3.3/10,000) compared to Georgia's (2.1/10,000) and Hawaii's (2.2/10,000) is probably due to genuinely higher birth prevalence of this defect in our population. Although this disease is inherited, environmental factors may be responsible for sporadic cases [45].

The birth prevalences of unilateral and bilateral renal agenesis have been reported at 10/10,000 and 2.5/10,000 respectively [46]. At the same time, a study on the epidemiology of kidney malformations reported marked variations in the birth prevalence of renal agenesis (0.6–29 per 10,000), which is considered to be mainly due to variations in the diagnostic procedures used [47]. Our overall figure (3.3/10, 000) was somewhat lower than the one for Georgia (4.4/10,000), while Finland reports a very low birth prevalence of this condition (0.7/10,000) in relation to a high rate of terminations of pregnancies with it: the rate for these defects increases to 2.43/10,000 when terminations are considered. We had no cases of bladder exstrophy; birth prevalences of this condition were also low in Finland (0.2/10,000) and Georgia (0.1/10,000).

## Conclusion

Our study, limited to selected major birth defects, is the first to provide population-based data on the prevalence rates at birth of congenital anomalies in defined populations in north-western Italy.

The methods used for data collection and case generation are proven to be ones that incorporate ample quality checks. Comparison with an independent source (birth certificates) suggests good completeness and quality. However in order to obtain a dataset that is as complete as possible, we plan to start using birth certificates as an additional data source. We expect to begin registering defects in elective terminations in the near future, as many defects are now identified prenatally and the exclusion of aborted cases complicates the identification of environmental factors in the etiology of these conditions [48]. As many malformations are compatible with life, particularly with modern treatments and surgical correction, they are not detected by vital registration systems. Mortality data cannot provide reliable indications of birth prevalence of live birth malformations and thus, registries of such malformations are important.

## Competing interests

The author(s) declare that they have no competing interests.

## Authors' contributions

G. Tagliabue conceived the study, participated in its design, verified diagnoses and complete case ascertainment, performed the analyses, and drafted the manuscript.

P. Contiero participated in conceiving, designing and analyzing the study, oversaw data collection and record linkage and reviewed the manuscript.

S. Fabiano, A. Maghini, A. Tittarelli performed automated data acquisition and standardization, and executed record linkage between the information sources.

E. Frassoldi, E. Costa, D. Gada abstracted clinical information from clinical records.

D. Vergani, M. Bellotti performed quality checks on the diagnoses generated by the defects registry.

R. Tessandori, S. Pisani, M. Gambino, F. Caramaschi, P. Crosignani performed a critical evaluation of the analysis and of the study design.

All authors have read and approved the final manuscript.

## References

[B1] Monitoring Birth Defects. http://www.cdc.gov/ncbddd/bd/monitoring.htm.

[B2] EUROCAT, European Network of Congenital Anomaly Registers. http://www.eurocat.ulster.ac.uk.

[B3] Petrini J, Damus K, Russell R, Poschman K, Davidoff MJ, Mattison D (2002). Contribution of birth defects to infant mortality in the United States. Teratology.

[B4] Istituto Superiore di Sanità, Ufficio di Statistica (1998). La mortalità per causa in Italia: 1980–1998. Roma, Italy.

[B5] Kalter H, Warkany J (1983). Medical progress. Congenital malformations: etiologic factors and their role in prevention (first of two parts). N Engl J Med.

[B6] Kalter H, Warkany J (1983). Congenital malformations (second of two parts). N Engl J Med.

[B7] Nelson K, Holmes LB (1989). Malformations due to presumed spontaneous mutations in newborn infants. N Engl J Med.

[B8] Kold Jensen T (2002). Congenital malformations, in: Children's health and environment: A review of evidence. A joint report from the European Environment Agency and the WHO Regional Office for Europe Office for Official Publications of the European communities.

[B9] Sekhobo JP, Druschel CM (2001). An evaluation of congenital malformations surveillance in New York State: an application of Centers for Disease Control and Prevention (CDC) guidelines for evaluating surveillance systems. Public Health Rep.

[B10] Rasmussen SA, Olney RS, Holmes LB, Lin AE, Keppler-Noreuil KM, Moore CA (2003). National Birth Defects Prevention Study. Guidelines for case classification for the National Birth Defects Prevention Study. Birth Defects Res A Clin Mol Teratol.

[B11] Surveillance and Research. http://www.icbd.org.

[B12] Tagliabue G, Maghini A, Fabiano S, Tittarelli A, Frassoldi E, Costa E, Nobile S, Codazzi T, Crosignani P, Tessandori R, Contiero P (2006). Consistency and accuracy of diagnostic cancer codes generated by automated registration: comparison with manual registration. PopulHealth Metr.

[B13] P Contiero, A Tittarelli, A Maghini, S Fabiano, E Frassoldi, E Costa, Gada D, Codazzi T, Crosignani P, Tessandori R, Tagliabue G (2007). Comparison with manual registration reveals satisfactory completeness and efficiency of a computerized cancer registration system. J biomedical Inform.

[B14] Contiero P, Evangelista A, Tittarelli A, Del Sette D, Krogh V, Berrino F, Tagliabue G (2002). Benign neoplasms: a follow-up study in Italy, 1993–1998. IARC Sci Publ.

[B15] Evangelista A, Tagliabue G, Del Sette D, Tittarelli A, Contiero P, Krogh V, Crosignani P, Berrino F (2002). Malignant tumour follow-up in Italy, 1993–1998. IARC Sci Publ.

[B16] Tagliabue G, Evangelista A, Tittarelli A, Del Sette D, Contiero P, Crosignani P, Berrino F, Micheli A (2002). Follow-up of the ORDET cohort, Lombardy Cancer Registry, 1987–1997. IARC Sci Publ.

[B17] Contiero P, Tittarelli A, Tagliabue G, Maghini A, Fabiano S, Crosignani P, Tessandori R (2005). The EpiLink record linkage software: presentation and result of linkage test on cancer registry files. Methods Inf Med.

[B18] http://www.nbdpn.org/current/resources/sgm/NBDPN_Guidelines.pdf.

[B19] Brenner H (1995). Use and limitations of the capture-recapture method in disease monitoring with two dependent sources. Epidemiology.

[B20] Monteagudo A, Tarricone NJ, Timor-Tritsch IE, Lerner JP (1996). Autosomal dominant cataracts of the fetus: early detection by transvaginal ultrasound. Ultrasound Obstet Gynecol.

[B21] Hernanz-Schulman M (2003). Infantile hypertrophic pyloric stenosis. Radiology.

[B22] (1996). Diagnostic testing for Prader-Willi and Angleman syndromes: Report of the ASHG/ACMG Test and Technology Transfer Committee. Am J Hum Genet.

[B23] (2002). Congenital Malformations Surveillance Report. A report from the National Birth Defects Prevention Network. Teratology.

[B24] Olsen CL, Polan AK, Cross PK (1996). Case ascertainment for state-based birth defects registries: characteristics of unreported infants ascertained through birth certificates and their impact on registry statistics in New York state. Paediatr Perinat Epidemiol.

[B25] Honein MA, Paulozzi LJ (1999). Birth defects surveillance: assessing the "gold standard". Am J Public Health.

[B26] Khoshnood B, De Vigan C, Vodovar V, Goujard J, Lhomme A, Bonnet D, Goffinet F (2005). Trends in prenatal diagnosis, pregnancy termination, and perinatal mortality of newborns with congenital heart disease in France, 1983–2000: a population-based evaluation. Pediatrics.

[B27] Hoffman JI, Kaplan S (2002). The incidence of congenital heart disease. J Am Coll Cardiol.

[B28] Botto LD, Correa A, Erickson JD (2001). Racial and temporal variations in the prevalence of heart defects. Pediatrics.

[B29] Pradat P, Francannet C, Harris JA, Robert E (2003). The epidemiology of cardiovascular defects, part I: a study based on data from three large registries of congenital malformations. Pediatr Cardiol.

[B30] Dolk H, Vrijheid M, Scott JE, Addor MC, Botting B, de Vigan C, de Walle H, Garne E, Loane M, Pierini A, Garcia-Minaur S, Physick N, Tenconi R, Wiesel A, Calzolari E, Stone D (2004). Toward the effective surveillance of hypospadias. Environ Health Perspect.

[B31] Pierik FH, Burdorf A, Nijman JM, de Muinck Keizer-Schrama SM, Juttmann RE, Weber RF (2002). A high hypospadias rate in The Netherlands. Hum Reprod.

[B32] Baskin LS, Himes K, Colborn T (2001). Hypospadias and endocrine disruption: is there a connection?. Environ Health Perspect.

[B33] Sharpe RM, Skakkebaek NE (1993). Are oestrogens involved in falling sperm counts and disorders of the male reproductive tract?. Lancet.

[B34] Sultan C, Balaguer P, Terouanne B, Georget V, Paris F, Jeandel C, Lumbroso S, Nicolas J (2001). Environmental xenoestrogens, antiandrogens and disorders of male sexual differentiation. Mol Cell Endocrinol.

[B35] Roizen NJ, Patterson D (2003). Down's syndrome. Lancet.

[B36] Allen WP, Stevenson RE, Thompson SJ, Dean JH (1996). The impact of prenatal diagnosis on NTD surveillance. Prenat Diagn.

[B37] Wong LY, Paulozzi LJ (2001). Survival of infants with spina bifida: a population study, 1979–94. Paediatr Perinat Epidemiol.

[B38] Honein MA, Paulozzi LJ, Mathews TJ, Erickson JD, Wong LY (2001). Impact of folic acid fortification of the US food supply on the occurrence of neural tube defects. JAMA.

[B39] Romitti PA, Lidral AC, Munger RG, Daack-Hirsch S, Burns TL, Murray JC (1999). Candidate genes for nonsyndromic cleft lip and palate and maternal cigarette smoking and alcohol consumption: evaluation of genotype-environment interactions from a population-based case-control study of orofacial clefts. Teratology.

[B40] Nyberg DA, Sickler GK, Hegge FN, Kramer DJ, Kropp RJ (1995). Fetal cleft lip with and without cleft palate: US classification and correlation with outcome. Radiology.

[B41] Zeiger JS, Beaty TH (2002). Is there a relationship between risk factors for oral clefts?. Teratology.

[B42] Gorski SM, Adams KJ, Birch PH, Friedman JM, Goodfellow PJ (1992). The gene responsible for X-linked cleft palate (CPX) in a British Columbia native kindred is localized between PGK1 and DXYS1. Am J Hum Genet.

[B43] Shaw GM, Nelson V, Carmichael SL, Lammer EJ, Finnell RH, Rosenquist TH (2002). Maternal periconceptional vitamins: interactions with selected factors and congenital anomalies?. Epidemiology.

[B44] Cragan JD, Martin ML, Moore CA, Khoury MJ (1993). Descriptive epidemiology of small intestinal atresia, Atlanta, Georgia. Teratology.

[B45] Hofstra RM, Osinga J, Buys CH (1997). Mutations in Hirschsprung disease: when does a mutation contribute to the phenotype. Eur J Hum Genet.

[B46] Hitchcock R, Burge DM (1994). Renal agenesis: an acquired condition?. J Pediatr Surg.

[B47] Harris J, Robert E, Kallen B (2000). Epidemiologic characteristics of kidney malformations. Eur J Epidemiol.

[B48] Limb CJ, Holmes LB (1994). Anencephaly: changes in prenatal detection and birth status, 1972 through 1990. Am J Obstet Gynecol.

